# Flux Growth, Crystal Structures, and Electronic Properties of the Ternary Intermetallic Compounds Ca_3_Pd_4_Bi_8_ and Ca_3_Pt_4_Bi_8_

**DOI:** 10.1021/acs.inorgchem.2c01248

**Published:** 2022-06-15

**Authors:** Alexander Ovchinnikov, Anja-Verena Mudring

**Affiliations:** † Department of Materials and Environmental Chemistry, Stockholm University, Svante Arrhenius väg 16 C, 10691 Stockholm, Sweden; ‡ Department of Chemistry, Aarhus University, Langelandsgade 140, 8000 Aarhus C, Denmark

## Abstract

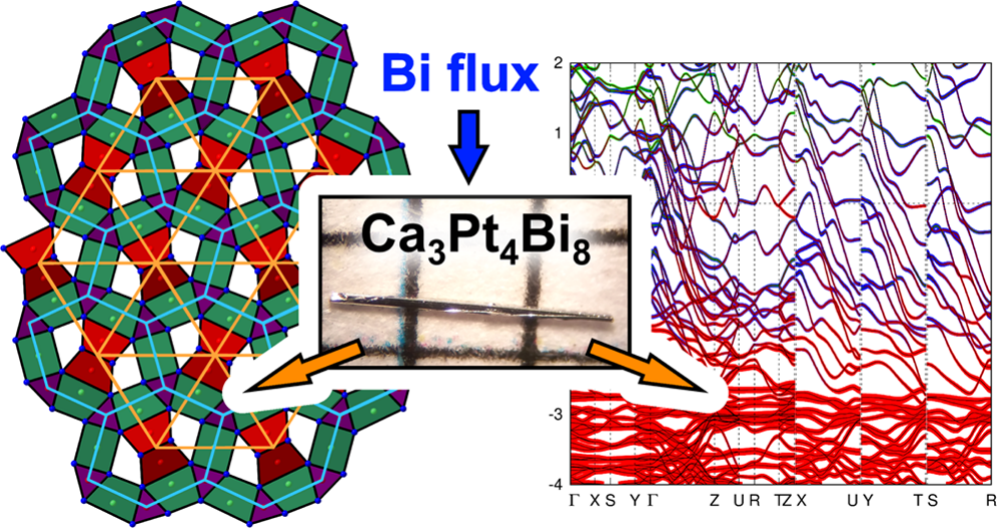

Reaction of the elements yielded Ca_3_Pt_4_Bi_8_ and CaPtBi, which are, to the best of our knowledge, the first reported ternary Ca–Pt–Bi compounds. The compounds crystallize isostructural to the Pd analogs Ca_3_Pd_4_Bi_8_ (own structure type) and CaPdBi (TiNiSi structure type), respectively. Employing a multistep temperature treatment allows for the growth of mm-sized single crystals of Ca_3_Pd_4_Bi_8_ and Ca_3_Pt_4_Bi_8_ from a Bi self-flux. Their crystal structures can be visualized as consisting of a three-dimensional extended polyanion [M_4_Bi_8_]^6–^ (M = Pd, Pt), composed of interlinked M–Bi chains propagating along the *c* direction, and Ca^2+^ cations residing in one-dimensional channels between the chains. First-principles calculations reveal quasi-one-dimensional electronic behavior with reduced effective electron masses along [001]. Bader analysis points to a strong anionic character of the M species (M = Pd, Pt) in Ca_3_M_4_Bi_8_. Thus, it is more appropriate to address the compounds Ca_3_Pd_4_Bi_8_ and Ca_3_Pt_4_Bi_8_ as a palladide and platinide, respectively. Magnetization measurements indicate diamagnetic behavior with no indications for superconductivity down to 2 K. Electrical resistivity data are consistent with metallic behavior and suggest predominant electron–phonon scattering.

## Abstract 2

Reaction of the elements yielded Ca_3_Pt_4_Bi_8_ and CaPtBi, which are, to the best of our knowledge, the first reported ternary Ca−Pt−Bi compounds.

## Introduction

Intermetallic compounds containing noble metals, such as Pd, Pt, or Au, are of special interest for condensed matter research. Their crystal structures, physical and chemical properties are often affected by the pronounced relativistic influences, leading to peculiar bonding situations. Relativistic stabilization of the *s* valence orbitals yields comparatively high electron affinities and, consequently, high electronegativities, resulting in compositions with strongly polarized bonding. With its high electronegativity, Au (2.54 on the Pauling scale^1^) is able to form an ionic intermetallic compound (Cs^+^)(Au^–^), featuring an optical bandgap of 2.5 eV and crystallizing isotypic to CsCl.^2−5^ The ionic nature of this phase lends it a range of properties, typical for inorganic salts, such as the formation of solvates, *e.g.*, CsAu·NH_3_.^6^

Even compounds with metallic behavior often display properties originating from the high electron affinity of the noble metal atoms. For instance, the binary compound Yb_3_Pd_5_ hosts Yb in the oxidation state +3, owing to the high electronegativity of Pd (2.20 on the Pauling scale),^7^ while in many intermetallic materials with less electronegative metals, Yb is found to be divalent.^8,9^ The Pd species in Yb_3_Pd_5_ assume therefore anionic character. In PdGa, partial charge transfer from Ga onto the electronegative Pd atoms is responsible for significant modification of the Pd(*d*) states compared to the elemental Pd, which contributes to a good performance of this material as a catalyst for the semihydrogenation of acetylene.^10^

Sizeable spin–orbit coupling, another effect of relativity, in compounds of Au and Pt often favors realization of nontrivial electronic topologies, such as the predicted three-dimensional Dirac semimetallic state in YbAuSb.^11^ Combination of noble metals with heavy *p*-elements provides even more opportunities for introduction of relativistic effects and targeting materials with unusual electronic properties. Thus, the binary compound of platinum with a heavy tetrel (group 14 element), Pb–PtPb_4_—has been recently identified as a potential Weyl-semimetal superconductor candidate. The superconducting transition temperature (*T*_c_) of PtPb_4_ is about 2.8 K.^12^ Another example is the Pd compound with a heavy pnictogen (group 15 element), Bi–PdBi—which exhibits Dirac surface states in the presence of superconductivity (*T*_c_ = 3.8 K).^13^ In general, superconducting properties are often observed for intermetallic phases containing noble metals and heavy *p*-elements. Besides the already-mentioned examples, a notable material is Au_2_Bi, which was one of the first intermetallic compounds discovered to be superconducting (*T*_c_ = 1.9 K).^14,15^

In the search for new superconductors with noble metals and *p*-elements, we focused our attention on the Ca–Pt–Bi and Ca–Pd–Bi ternary systems. By careful optimization of the crystal growth procedure, high-quality single crystals of the new compound Ca_3_Pt_4_Bi_8_ and its known isostructural analog Ca_3_Pd_4_Bi_8_ have been produced. Although both materials lack bulk superconductivity down to 2 K, they do demonstrate interesting electronic properties, namely, quasi-one-dimensional electronic transport, as predicted by our first-principles calculations. The high electronegativity of the noble metals, M = Pd, Pt, results in the pronounced anionic character of the M species in Ca_3_M_4_Bi_8_.

## Experimental Section

### Synthesis

All weighing and mixing procedures were performed in an Ar-filled glovebox with H_2_O and O_2_ levels below 0.1 ppm.

**Figure 1:**
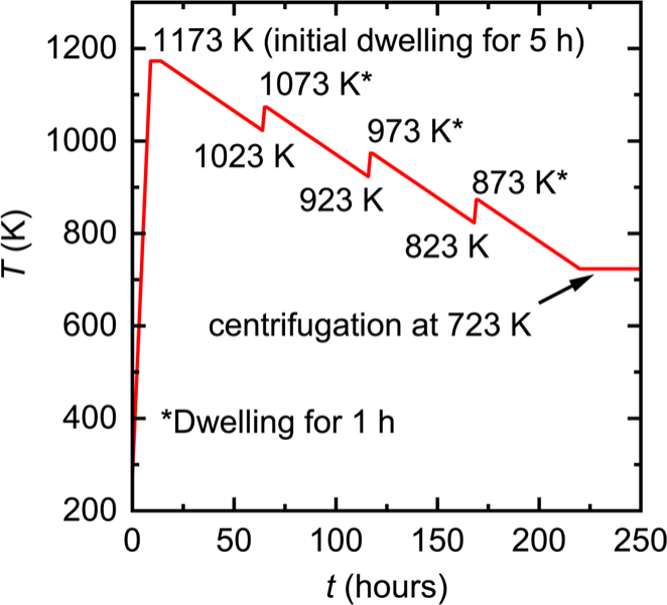
Schematic representation of the temperature profile used for the crystal growth of Ca_3_M_4_Bi_8_ (M = Pd, Pt).

Needle-like single crystals of Ca_3_M_4_Bi_8_ (M = Pd, Pt) with axial dimensions of up to several mm were grown from a liquid Bi flux. Metallic Ca (Alfa Aesar), M (Alfa Aesar and Neyco for M = Pd and Pt, respectively), and Bi (Alfa Aesar), all with purity ≥99.98 wt %, were mixed in the ratio Ca/M/Bi = 3:4:12 and placed in an alumina crucible topped with quartz wool. The crucible was loaded in a fused silica tube, which was sealed under vacuum. The temperature program used for reaction and crystal growth is sketched in Figure 1. The reactor was first heated to 1173 K in a box furnace at a rate of 100 K/h and kept at this temperature for 5 h. After this homogenization step, a complex sawtooth-shaped temperature profile was applied, containing several cooling and heating steps. Such treatment was found to promote the growth of larger crystals, as will be explained in the Results and Discussion section (*vide infra*). In every cooling step, the temperature was lowered by 150 K at a rate of 3 K/h. In the heating steps, the temperature was increased by 50 K at a rate of 50 K/h and kept at the new *T* value for 1 h before proceeding to the next cooling step. After reaching a temperature of 723 K, the reactor was taken out of the furnace, flipped over, and placed in a centrifuge to remove the excess liquid Bi. The grown crystals were isolated mechanically from the quartz wool filter after break-opening the fused silica tube.

In addition to flux synthesis and growth, we attempted examination of the phase formation in the Ca–Pt–Bi system employing high-temperature annealing of the elements in Nb tubes. For this purpose, the elements were mixed in different ratios, gently pressed to form a pellet, and loaded in a metal tube, sealed at one end. The metal tube was then transported to a custom-built arc-welding facility and sealed under about 600 mbar of high-purity Ar. After that, the sealed containers were enclosed in evacuated fused silica tubes. The reaction mixtures were heated to 1173 K at a rate of 200 K/h, kept at this temperature for 48 h, and cooled to room temperature at a rate of 5 K/h.

### Powder X-ray Diffraction (PXRD)

PXRD measurements were carried out on two powder diffractometers: Bruker Phaser D2 diffractometer (filtered Cu Kα radiation, λ_mean_ = 1.5418 Å), which was mainly utilized for phase identification, and PANalytical X’Pert diffractometer (monochromatized Cu Kα1 radiation, λ = 1.54056 Å), employed for high-quality data collections used in crystal structure refinements. In both cases, the data were acquired in the reflection geometry in the 2Θ range of 5–90°. The ground powders were dusted onto low-background silicon holders through a 60 μm sieve. The sieving procedure helped reduce the preferred orientation; although, due to the inherently anisotropic shape of the grown crystals, texture effects were still pronounced in the PXRD patterns. Rietveld refinements were performed with the Jana2006 software.^16^

### Single-Crystal X-ray Diffraction (SCXRD)

Single crystals were cut to desired dimensions with a scalpel under vacuum grease. The crystals were attached to low-background MiTeGen plastic loops and placed on a Bruker D8 Venture diffractometer (Mo Kα, λ = 0.71073 Å) equipped with a PHOTON 100 CMOS detector. All data were collected at room temperature. Data integration and absorption corrections were accomplished with the SAINT^17^ and SADABS^18^ software, respectively. Crystal structures were solved by dual-space methods as implemented in SHELXT^19^ and refined by a full-matrix least-squares method on *F*^2^ with SHELXL.^20^ Atomic coordinates were standardized using STRUCTURE TIDY.^21^ Details of the data collection and selected crystallographic parameters are summarized in Tables 1–3.

**Table 1:** Refinement Details and Selected Crystallographic Data for Ca_3_*M*_4_Bi_8_ (*M* = Pd, Pt, Space Group *Pbam*, Room Temperature, Mo Kα λ = 0.71073 Å, *Z* = 2)

refined composition	Ca_3_Pd_4_Bi_8_	Ca_3_Pt_4_Bi_8_
CCDC no.	2165536	2165537
fw (g mol^–1^)	2217.68	2572.44
*a* (Å)	10.8345(10)	10.8683(6)
*b* (Å)	17.0788(14)	17.0386(9)
*c* (Å)	4.1526(4)	4.1433(2)
*V* (Å^3^)	768.40	767.26
ρ_calc_ (g cm^–3^)	9.58	11.13
μ_Mo Kα_ (mm^–1^)	96.7	128.6
*R*_int_	0.046	0.040
*R*_1_ [*I* > 2σ(*I*)]^a^	0.024	0.022
w**R**_2_ [*I* > 2σ(*I*)]^a^	0.047	0.049
*R*_1_ [all data]^a^	0.027	0.024
w**R**_2_ [all data]^a^	0.048	0.050
Δρ_max,min_ (e Å^–3^)	1.44, −2.29	2.13, −1.90

a *R*_1_ = ∑||*F*_o_| – |*F*_c_||/∑|*F*_o_|; *w*R**_2_ = [∑[*w*(*F*_o_^2^ – *F*_c_^2^)^2^]/ ∑[w(*F*_o_^2^)^2^]]^1/2^, where *w* = 1/[σ^2^*F*_o_^2^ + (*AP*)^2^ + (*BP*)] and *P* = (*F*_o_^2^ + 2*F*_c_^2^)/3. *A* and *B* are the respective weight coefficients (see the CIF data).

**Table 2:** Atomic Coordinates and Equivalent Isotropic Displacement Parameters (Å^2^) for Ca_3_*M*_4_Bi_8_ (*M* = Pd, Pt)

atom	site	*x*	*y*	*z*	*U*_eq_^a^
**Ca**_**3**_**Pd**_**4**_**Bi**_**8**_					
Ca1	4g	0.3576(2)	0.19902(15)	0	0.0214(5)
Ca2	2a	0	0	0	0.0301(9)
Pd1	4h	0.45378(9)	0.07844(6)	1/2	0.0255(2)
Pd2	4g	0.07146(10)	0.17575(6)	0	0.0244(2)
Bi1	4h	0.11000(4)	0.28664(3)	1/2	0.02203(12)
Bi2	4h	0.19577(4)	0.08586(3)	1/2	0.02193(12)
Bi3	4h	0.39122(5)	0.35709(3)	1/2	0.02377(12)
Bi4	4g	0.12981(4)	0.46110(3)	0	0.02417(12)
**Ca**_**3**_**Pt**_**4**_**Bi**_**8**_					
Ca1	4g	0.3638(2)	0.19842(14)	0	0.0200(4)
Ca2	2a	0	0	0	0.0269(7)
Pt1	4h	0.45488(4)	0.08008(2)	1/2	0.02151(11)
Pt2	4g	0.07816(4)	0.17588(2)	0	0.01901(11)
Bi1	4h	0.11464(3)	0.28720(2)	1/2	0.01749(10)
Bi2	4h	0.19848(4)	0.08531(2)	1/2	0.01755(11)
Bi3	4h	0.39627(4)	0.35697(2)	1/2	0.01950(11)
Bi4	4g	0.12981(4)	0.46039(2)	0	0.02055(11)

a *U*_eq_ is defined as one-third of the trace of the orthogonalized *U_ij_* tensor.

**Table 3:** Selected Interatomic Distances (Å) for Ca_3_M_4_Bi_8_ (M = Pd, Pt)

atoms		distances (M = Pd)	distances (M = Pt)
Ca1	–M1 × 2	3.104(2)	3.0556(17)
	–M2	3.125(3)	3.128(2)
	–M2	3.153(3)	3.165(2)
	–Bi2 × 2	3.335(2)	3.3518(17)
	–Bi3 × 2	3.425(2)	3.4225(19)
	–Bi1 × 2	3.442(2)	3.4326(18)
	–Bi1 × 2	3.708(2)	3.7302(19)
Ca2	–M2 × 2	3.0999(10)	3.1147(4)
	–Bi2 × 4	3.3106(4)	3.3253(3)
	–Bi3 × 4	3.4143(4)	3.3915(3)
M1	–M1	2.861(2)	2.8997(8)
	–Bi2	2.7982(12)	2.7880(6)
	–Bi1	2.8590(11)	2.8510(6)
	–Bi4 × 2	2.8990(8)	2.8951(4)
	–Bi4 × 2	3.0245(8)	3.0491(4)
M2	–Bi1 × 2	2.8411(8)	2.8368(4)
	–Bi3 × 2	2.9050(8)	2.9177(4)
	–Bi2 × 2	2.9124(8)	2.8953(4)
Bi1	–Bi3	3.2758(7)	3.2835(6)
	–Bi3	3.4123(7)	3.4157(6)
	–Bi2	3.5528(7)	3.5586(6)
	–Bi4 × 2	3.6379(6)	3.6093(5)
Bi2	–Bi3	3.4406(7)	3.4287(6)
	–Bi4 × 2	3.5245(6)	3.5078(5)
	–Bi1	3.5528(7)	3.5586(6)
Bi3	–Bi1	3.2758(7)	3.2835(6)
	–Bi1	3.4123(7)	3.4157(6)
	–Bi2	3.4406(7)	3.4287(6)
Bi4	–Bi4	3.1109(10)	3.1277(8)
	–Bi2 × 2	3.5245(6)	3.5078(5)
	–Bi1 × 2	3.6379(6)	3.6093(5)

### Physical Property Measurements

The surface of the crystals was cleaned with a scalpel to remove traces of the Bi flux before the measurements.

Magnetization was measured on a Quantum Design Physical Property Measurement System (PPMS) in the temperature range *T* = 2–300 K under applied fields of 20–1000 Oe, using the vibrating-sample magnetometer option (VSM). The samples were ground and packed in polypropylene containers. Isothermal magnetization measurements were taken at 2 K under applied fields of up to 6 T employing the same setup. The contribution of the sample holder was measured separately and subtracted from the signal of the examined materials.

The temperature dependence of the electrical resistivity was recorded using a four-probe setup on the PPMS in the temperature range of 2–300 K. Pt wires with a diameter of 25 μm were attached to the crystal surface with a PELCO conductive silver paint. Measurements were performed on cooling and heating to assure reproducibility.

### First-Principles Calculations

Electronic structures of Ca_3_Pd_4_Bi_8_ and Ca_3_Pt_4_Bi_8_ were evaluated within the density functional theory approach (DFT) using the TB-LMTO-ASA code on the scalar-relativistic level.^22^ The von Barth–Hedin flavor of the local density approximation functional (LDA) was applied.^23^ The basis set contained the following states: Ca: 4s, (4p), 3d; Pd: 5s, 5p, 4d, (4f); Pt: 6s, 6p, 5d, (5f); and Bi: 6s, 6p, (6d), (5f), with the states in parenthesis being down-folded. The Brillouin zone was sampled by a 6 × 4 × 12 *k*-point grid. To satisfy the atomic sphere approximation (ASA), empty spheres were added following the standard procedure in the LMTO code. The chemical bonding was analyzed by means of the Crystal Orbital Hamilton Population approach (COHP).^24^ Bader charges were calculated by integration of the total charge density with the program Critic2.^25^

## Results and Discussion

### Synthesis

Our original motivation for the exploratory studies in the Ca–Pt–Bi system was the search for possible ternary superconducting materials. Some compounds containing Pt–Bi polyanions have been found to be superconducting at low temperatures, including LuPtBi^26^ and BaPt_2_Bi_2_.^27^ However, no ternary compounds have been reported in the Ca–Pt–Bi system, which raises questions, as whether any such compounds exist and whether they display superconductivity as well.

In our early attempts to produce ternary Ca–Pt–Bi phases, we used high-temperature annealing of the elements in metal containers made of Nb. These attempts yielded two new ternary phases: Ca_3_Pt_4_Bi_8_ and CaPtBi, isostructural to the Pd analogs Ca_3_Pd_4_Bi_8_^28^ and CaPdBi.^29^ To the best of our knowledge, these are the first reported ternary Ca–Pt–Bi compounds. However, reactions in Nb tubes yielded multiphase samples, even when the correct starting compositions were used (Figure S1a). In particular, CaPtBi, crystallizing in the TiNiSi structure type, could not be prepared single-phase. However, the structure of this compound was determined from single-crystal X-ray diffraction data of a single crystal extracted from a sample with about 70 wt % of CaPtBi. This sample was synthesized in an Nb tube, as described in the Experimental Section, starting from the Ca:Pt:Bi = 1:1:1 element ratio. Crystallographic information for CaPtBi is provided in Tables S1 and S2. In short, CaPtBi crystallizes in the TiNiSi structure type, just like its Pd analog, CaPdBi.^29^ Details on the physical properties of CaPtBi will be communicated once a pure sample is available.

The failure to produce single-phase samples in Nb tubes is likely related to side reactions with the container material. Indeed, closer examination of the PXRD patterns and analysis of the single crystals revealed the occasional presence of the binary impurity Nb_3_Pt in the samples, especially in Pt-rich ones. This impurity was found to be responsible for the apparent superconductivity of most of the investigated samples, visible in the magnetization data below about 9 K (Figure S1b).

To avoid contamination by possible superconducting impurities and assure high purity of the synthesized materials, we attempted single-crystal growth of the newly discovered ternary Ca–Pt–Bi phases from metal fluxes. Although so far, this method has been proved unsuccessful for the production of CaPtBi, millimeter-sized single crystals of Ca_3_Pt_4_Bi_8_ could be obtained using the Bi flux approach (since Bi is also one of the constituent elements in the composition, the applied procedure can be called a “self-flux method”^30^).

In the first experiments, upon slow cooling (3–5 K/h) from a Ca–Pt–Bi mixture with excess Bi, numerous submillimeter-sized crystals of Ca_3_Pt_4_Bi_8_ were grown. Decreasing the cooling rate even further (down to 1.5 K/h) did not affect the size of the crystals significantly, suggesting that fast seed formation could be the reason for insufficiently large final crystals. Therefore, to improve the crystal size, we developed a multistep cooling procedure, with several cooling and heating segments. The idea behind was to dissolve smaller crystals in the heating steps, while preserving the larger ones as seed crystals, and to allow the latter to grow in the cooling segments, in a process similar to Ostwald ripening. Indeed, the developed technique enabled the growth of needle-like single crystals with lengths of up to about 7 mm.

Since we were interested in studying potential superconductivity, we also grew single crystals of the known isostructural compound Ca_3_Pd_4_Bi_8_ using the same flux growth approach. The electron–phonon coupling is expected to be stronger in materials with lighter atoms. Therefore, we anticipated that the Pd representative could be a better candidate for superconductivity. Nevertheless, as we will discuss below, neither Ca_3_Pt_4_Bi_8_, nor its Pd analog, exhibits superconductivity above 2 K.

**Figure 2:**
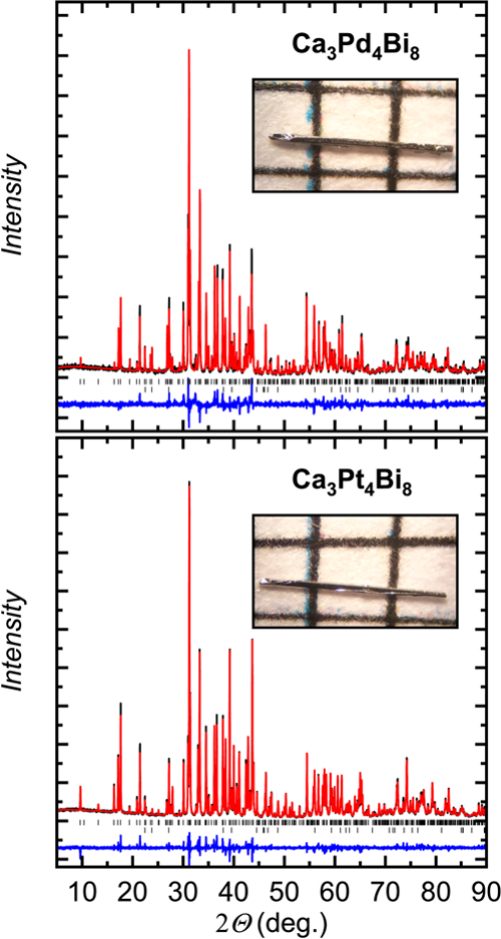
Powder X-ray diffraction patterns (Cu Kα1) along with the corresponding Rietveld refinement for Ca_3_Pd_4_Bi_8_ (top) and Ca_3_Pt_4_Bi_8_ (bottom). Experimental data, calculated profile, and difference curve are shown in black, red, and blue, respectively. Tick marks indicate the positions of the Bragg reflections for the major phase (upper row) and the residual Bi (bottom row). The refined Bi content is around 4 wt % in both samples. Insets show optical images of the respective flux-grown crystals on an mm grid.

Both Ca_3_Pd_4_Bi_8_ and Ca_3_Pt_4_Bi_8_ samples, produced by the self-flux method, demonstrate high purity, as indicated by PXRD analysis (Figure 2). The only detectable secondary phase is the residual Bi flux, occasionally present on the surface of the crystals.

### Crystal Structure

Analysis of the needle-like single crystals of Ca_3_Pd_4_Bi_8_ and Ca_3_Pt_4_Bi_8_ indicates that the two compounds are isostructural and crystallize in space group *Pbam* (No. 55). The unit cells exhibit relatively large *a* and *b* edges and a short *c* edge: for Ca_3_Pd_4_Bi_8_, *a* = 10.8345(10) Å, *b* = 17.0788(14) Å, *c* = 4.1526(4) Å; for Ca_3_Pt_4_Bi_8_, *a* = 10.8683(6) Å, *b* = 17.0386(9) Å, *c* = 4.1433(2) Å (as determined from the SCXRD data). Indexing of the crystal faces on the single-crystal diffractometer suggests that the crystalline needles grow along the [001] direction. The structure of Ca_3_Pd_4_Bi_8_ has been reported before.^28^ Therefore, we will focus on describing Ca_3_Pt_4_Bi_8_ and its specific features.

**Figure 3:**
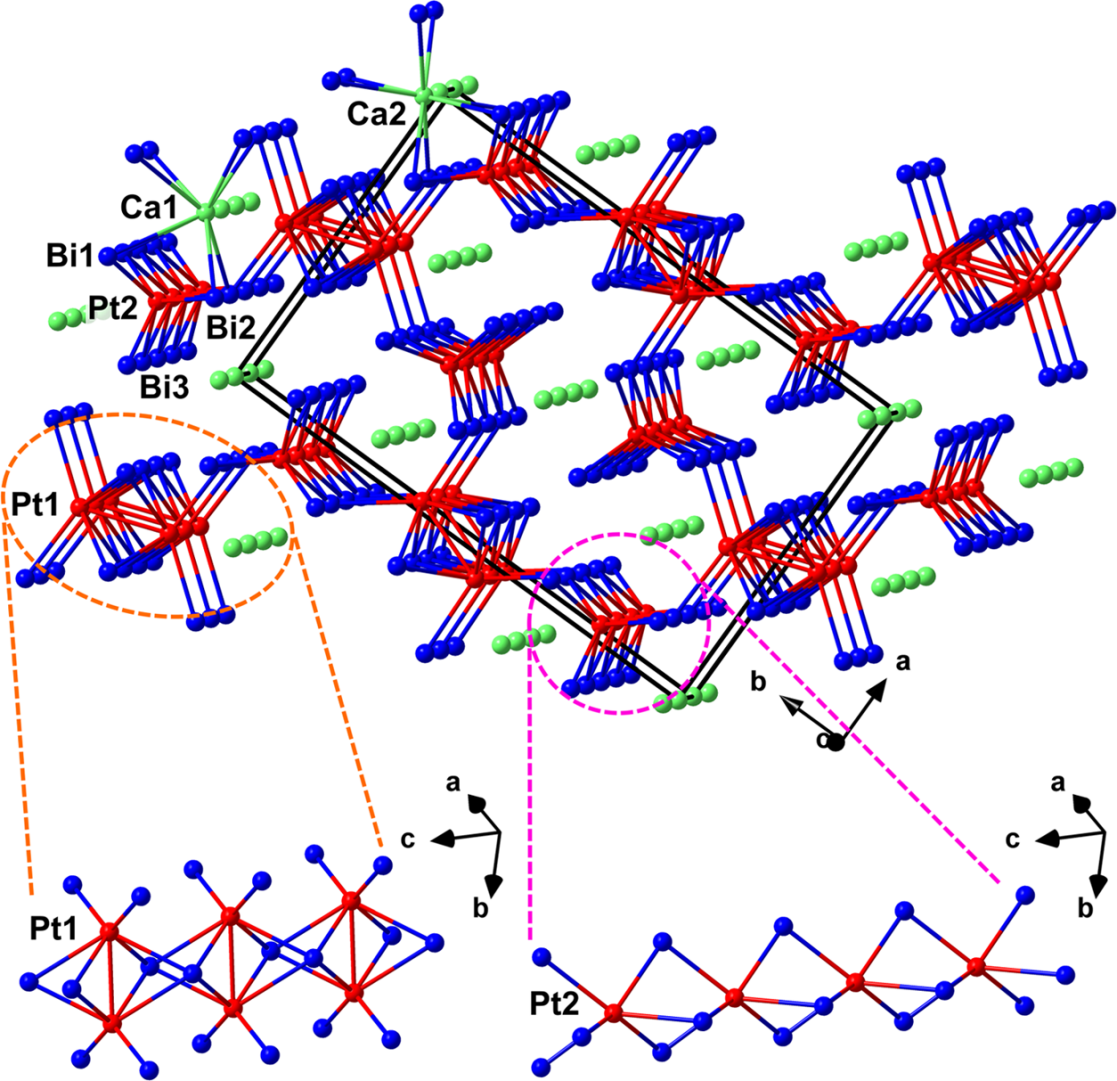
Crystal structure of Ca_3_Pt_4_Bi_8_ viewed as Ca atoms embedded in a Pt–Bi framework. The unit cell is outlined in black. The Bi coordinations around Ca and Pt atoms and Pt–Pt interatomic connections are shown. The close-up view of the polyhedral Pt–Bi chains constituting the framework is given at the bottom.

Considering high electronegativity differences between Ca (1.00 on the Pauling scale) and Pt (2.28) or Bi (2.02),^1^ the ionic notation (Ca^2+^)_3_[Pt_4_Bi_8_]^6–^ appears to be a valid approximation of the charge partitioning in Ca_3_Pt_4_Bi_8_, as we will also show using first-principles calculations (*vide infra*). Hence, the principal building block in the discussed crystal structure is the complex polyanion [Pt_4_Bi_8_]^6–^ (Figure 3). Although this structural unit is extended in all three dimensions, it can be viewed as consisting of two kinds of interlinked polyhedral Pt–Bi chains, running along the *c* direction, which lends a quasi-one-dimensional character to the structure (Figure 3, bottom).

The first kind of chains, accommodating Pt1 species, is built up of dimeric Pt_2_Bi_8_ units, representing pairs of face-sharing PtBi_6_-distorted trigonal prisms. These dimeric units, linked by edge-sharing into chains that run parallel to [001], are hallmarked by a relatively short Pt–Pt distance of 2.900 Å, suggesting metal–metal bonding.

The second type of chains is based on the same kind of coordination environment—a trigonal prism of Bi atoms around the Pt2 species. However, instead of forming dimers, the prisms link directly into chains parallel to [001] by face sharing. The shortest Pt–Pt contact in these chains measures 4.143 Å, which is clearly outside the range of typical bonding contacts.^31^

**Figure 4:**
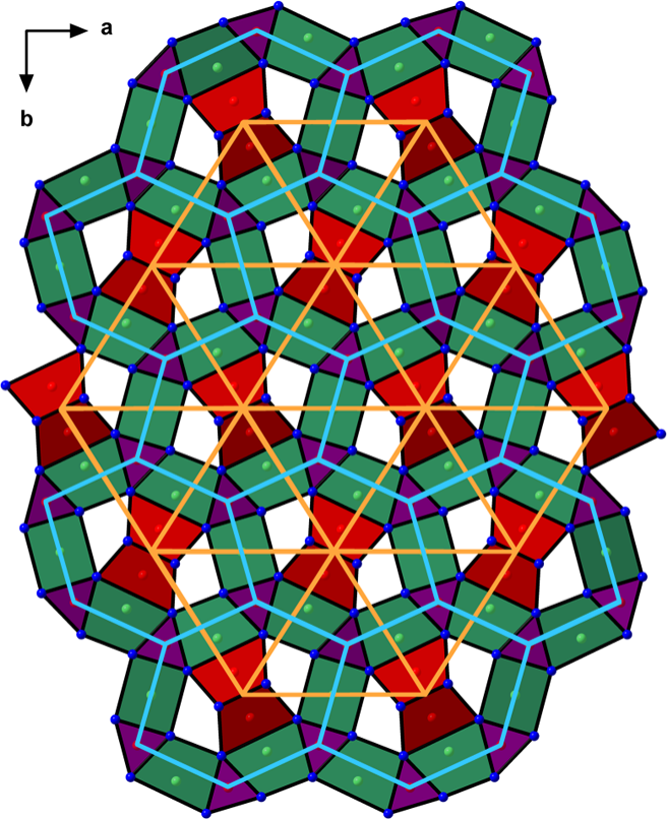
Polyhedral representation of the Ca_3_Pt_4_Bi_8_ crystal structure projected along [001]. Bi polyhedra around Ca (Ca1 + Ca2), Pt1, and Pt2 are shown in dark green, red, and violet, respectively. The distorted trigonal and honeycomb rod packings of the polyhedral Pt–Bi chains are highlighted in orange and light blue, respectively. Atom color codes: Ca: green, Pt: red, and Bi: blue.

The two types of polyhedral chains join by sharing Bi vertices. The chains propagate along the *c* direction and form two interpenetrating motifs: a distorted trigonal rod packing of the chains based on the Pt1 species and a distorted honeycomb rod packing of the chains based on the Pt2 species (Figure 4). The Pt–Bi distances in both types of chains span from 2.788 to 3.049 Å, in accordance with bonding Pt–Bi distances in other reported compounds.^27,32−35^

The Ca^2+^ ions occupy the channels formed between the Pt–Bi chains in the [Pt_4_Bi_8_]^6–^ framework. The Ca–Bi distances that can be considered bonding contacts lie in the region 3.325–3.730 Å, typical for Ca bismuthides.^36−40^ The symmetry-independent Ca1 and Ca2 positions are both eight-fold coordinated by Bi, adopting distorted cubic environments. The resulting CaBi_8_ polyhedra link into chains along [001] by face sharing. Just like the polyhedral chains accommodating the Pt2 species, the Ca–Bi chains are arranged in a distorted honeycomb rod packing (Figure 4).

It is worth noting that besides the Pt–Bi and Ca–Bi bonds, other bonding interactions that can be deduced based on the interatomic distances are Ca–Pt and Bi–Bi. The shortest Ca–Pt contacts fall in the range of 3.056–3.165 Å, which is similar to the distances found in Ca platinides, *i.e.*, compounds with anionic Pt species, such as CaPt_2_ and CaPt_5_.^41^ The Bi–Bi bonding is suggested by the presence of Bi–Bi contacts of 3.128–3.609 Å. All of these values appear to exceed single Bi–Bi bonds but are in line with multicenter bonding.^38−40,42−44^ Due to this complex chemical bonding pattern, the Bi–Bi interactions form an extended three-dimensional network (Figure S2), not very typical for polyanionic compounds of pnictogens (group 15 elements).^45,46^

A comparison of the Ca_3_M_4_Bi_8_ crystal structures (M = Pd, Pt) suggests slight variations in the atomic packing. Thus, despite the smaller unit cell volume, the structure of Ca_3_Pt_4_Bi_8_ exhibits a noticeably larger unit cell parameter *a*. This can be, in part, explained by an anisotropic elongation of some of the Ca–Bi bonding contacts in Ca_3_Pt_4_Bi_8_. The latter effect is in turn related to a more sizeable electron density transfer from the Ca atoms onto the M atoms for M = Pt, which calls for redistribution of the bond lengths in the Ca–Bi polyhedra. In this respect, it would be interesting to study the evolution of the unit cell parameters in the solid-solution Ca_3_Pd_4–*x*_Pt_*x*_Bi_8_, which can likely be prepared.

### Physical Properties

Since we were interested in realization of superconductivity in the Ca–M–Bi system with M = Pd or Pt, we examined the magnetic properties of Ca_3_Pd_4_Bi_8_ and Ca_3_Pt_4_Bi_8_. No Meissner effect was observed down to 2 K under applied fields as low as 20 Oe, suggesting the lack of bulk superconductivity.

It is worth noting that other Bi-containing superconductors with Pd or Pt often exhibit superconducting properties at temperatures outside the range of our measurements. Thus, a critical temperature of about 1.6 K was reported for the half-Heusler phases YPdBi and LuPdBi,^47^ while the isostructural Pt representatives YPtBi and LuPtBi superconduct below 0.8 and 1.0 K, respectively.^26,48^ The ternary Pt-bearing intermetallic compound BaPt_2_Bi_2_ displays superconductivity below 2.0 K.^27^ In contrast, the binary material PdBi_2_ enters a superconducting state at a relatively high temperature of 5.3 K,^49^ while the Bi-poorer PdBi superconducts below 3.8 K.^13^ The absence of superconductivity in Ca_3_Pd_4_Bi_8_ and Ca_3_Pt_4_Bi_8_ above 2 K does not exclude that the zero-resistance state can be observed at lower temperatures, which will require further studies.

**Figure 5:**
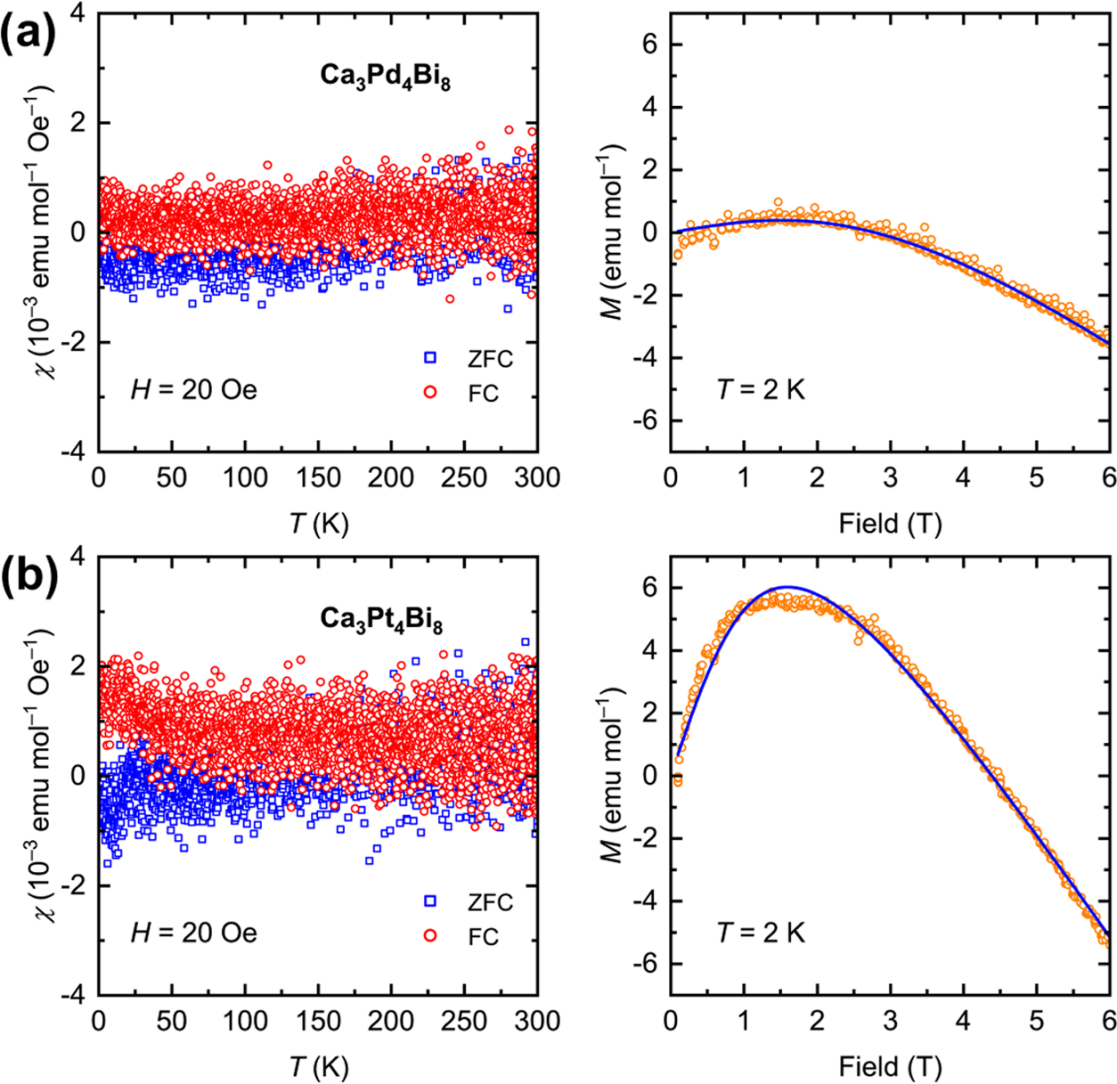
Temperature dependence of magnetic susceptibility (left) and isothermal magnetization at *T* = 2 K (right) for Ca_3_Pd_4_Bi_8_ (a) and Ca_3_Pt_4_Bi_8_ (b). ZFC and FC refer to zero-field-cooled and field-cooled data, respectively. Open orange circles denote experimental isothermal magnetization data and the blue solid curves correspond to the best nonlinear fits with eq 1.

The temperature dependence of the magnetic susceptibility (corrected for the sample holder contribution) for Ca_3_Pd_4_Bi_8_ and Ca_3_Pt_4_Bi_8_ measured under a field of 20 Oe is shown in Figure 5 (a,b, left). Both compounds demonstrate a weak signal, with essentially no temperature dependence, except for a small paramagnetic contribution, especially visible in the field-cooled data of Ca_3_Pt_4_Bi_8_.

Since the data at 20 Oe were noisy, it was not possible to conclude whether the studied materials are intrinsically diamagnetic or paramagnetic. To resolve this, we measured isothermal magnetization at 2 K under applied fields of up to 6 T. The data, presented in Figure 5 (a,b, right), suggest that the prevailing behavior is diamagnetic, although both compounds contain small amounts of paramagnetic impurities. To extract the intrinsic diamagnetic contribution, χ_int_, we fitted the isothermal magnetization data with the expression
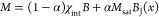
1where α is the mole fraction of the paramagnetic impurity and *M*_sat_ is the saturation magnetization for the paramagnetic impurity. The field dependence of the paramagnetic signal is described by the Brillouin function *B*_J_(*x*), *x* = *Jg*μ_B_*B*/*k*_B_*T*, where *J* is the total angular momentum, *g* is the *g*-factor, μ_B_ is the Bohr magneton, *k*_B_ is the Boltzmann constant, and *B* and *T* are the field and temperature, respectively.

The fits yield the intrinsic diamagnetic contributions χ_int_ = −1.568 10^–4^ emu mol^–1^ for Ca_3_Pd_4_Bi_8_ and χ_int_ = −3.336 10^–4^ emu mol^–1^ for Ca_3_Pt_4_Bi_8_. By taking the total electronic density of states (DOS) at the Fermi level from our first-principles calculations (*vide infra*), it is possible to estimate the core diamagnetism, χ_core_, using the formula
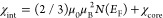
2where the first term takes into account the Pauli paramagnetism and Landau diamagnetism (χ_Landau_ = (−1/3)χ_Pauli_). μ_0,_ μ_B_, and *N*(*E*_F_) are the vacuum permeability, Bohr magneton, and DOS at the Fermi level, respectively.

With the calculated *N*(*E*_F_) = 5.04 states eV^–1^ f.u.^–1^ and 6.48 states eV^–1^ f.u.^–1^ for Ca_3_Pd_4_Bi_8_ and Ca_3_Pt_4_Bi_8_, respectively, the core diamagnetic susceptibility was estimated to be χ_core_(Ca_3_Pd_4_Bi_8_) = −2.654 10^–4^ emu mol^–1^ and χ_core_(Ca_3_Pt_4_Bi_8_) = −4.756 10^–4^ emu mol^–1^. The higher absolute value for the Pt member is expected owing to the larger number of core electrons in Pt in comparison to Pd.

**Figure 6:**
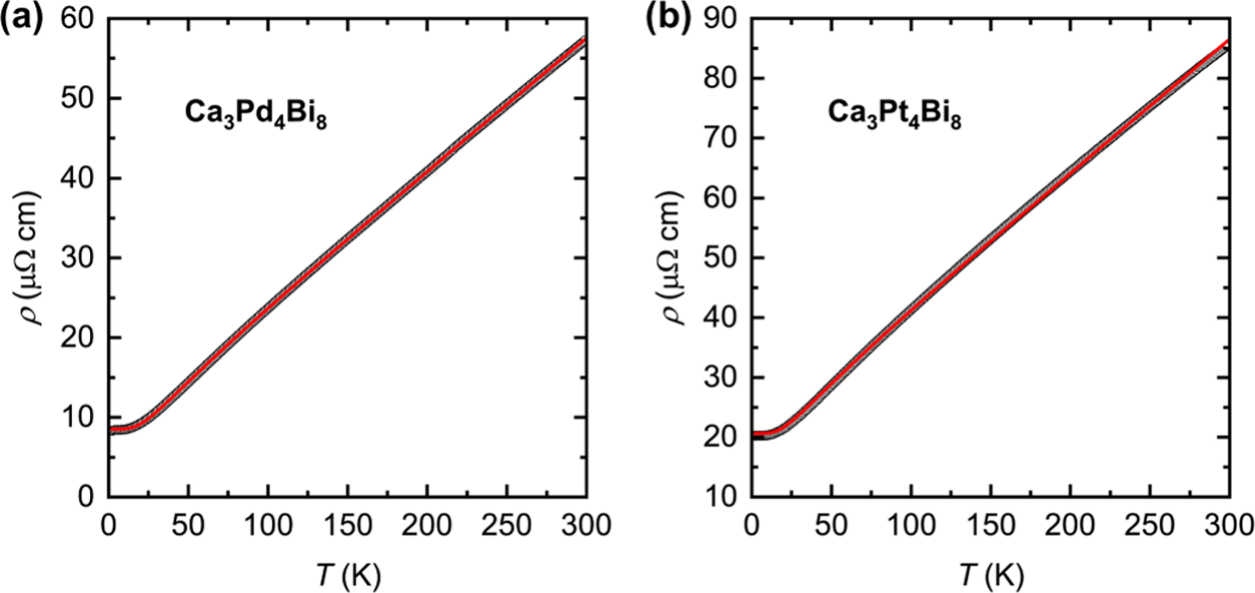
Temperature dependence of electrical resistivity for Ca_3_Pd_4_Bi_8_ (a) and Ca_3_Pt_4_Bi_8_ (b). Open circles denote experimental data and the blue red curves are the best nonlinear fits with eq 3.

The temperature dependence of the electrical resistivity, ρ(*T*), for Ca_3_Pd_4_Bi_8_ and Ca_3_Pt_4_Bi_8_ is shown in Figure 6. Due to the needle-like shape of the crystals, it was possible to measure the resistivity only along the needle axis, *i.e.*, along the [001] crystal direction. Since we expect anisotropic electron transport based on our first-principles calculations (*vide infra*), the resistivity is anticipated to be higher in perpendicular directions. Both compounds display metallic behavior with room temperature resistivity of about 57 μΩ cm (Ca_3_Pd_4_Bi_8_) and 86 μΩ cm (Ca_3_Pt_4_Bi_8_) and residual resistivity ratios, RRRs = ρ(300 K)/ρ(2 K), of 6.7 and 4.2, respectively. The temperature evolution of the resistivity can be well fitted with the modified Bloch–Grüneisen equation
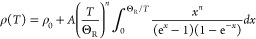
3where ρ_0_ is the residual resistivity, Θ_R_ is the Debye temperature, and *A* is a material-specific constant. The fits yield the values ρ_0_ = 8.5 μΩ cm, Θ_R_ =127.7 K, *A* = 76.7 μΩ cm, and *n* = 4.64 for Ca_3_Pd_4_Bi_8_ and ρ_0_ = 20.6 μΩ cm, Θ_R_ =116.6 K, *A* = 103.3 μΩ cm, and *n* = 4.99 for Ca_3_Pt_4_Bi_8_. The fitted power of *n* in the equation is close to 5 for both materials, as expected for metals with dominating electron–phonon scattering.^50^

### Electronic Structure and Chemical Bonding

**Figure 7:**
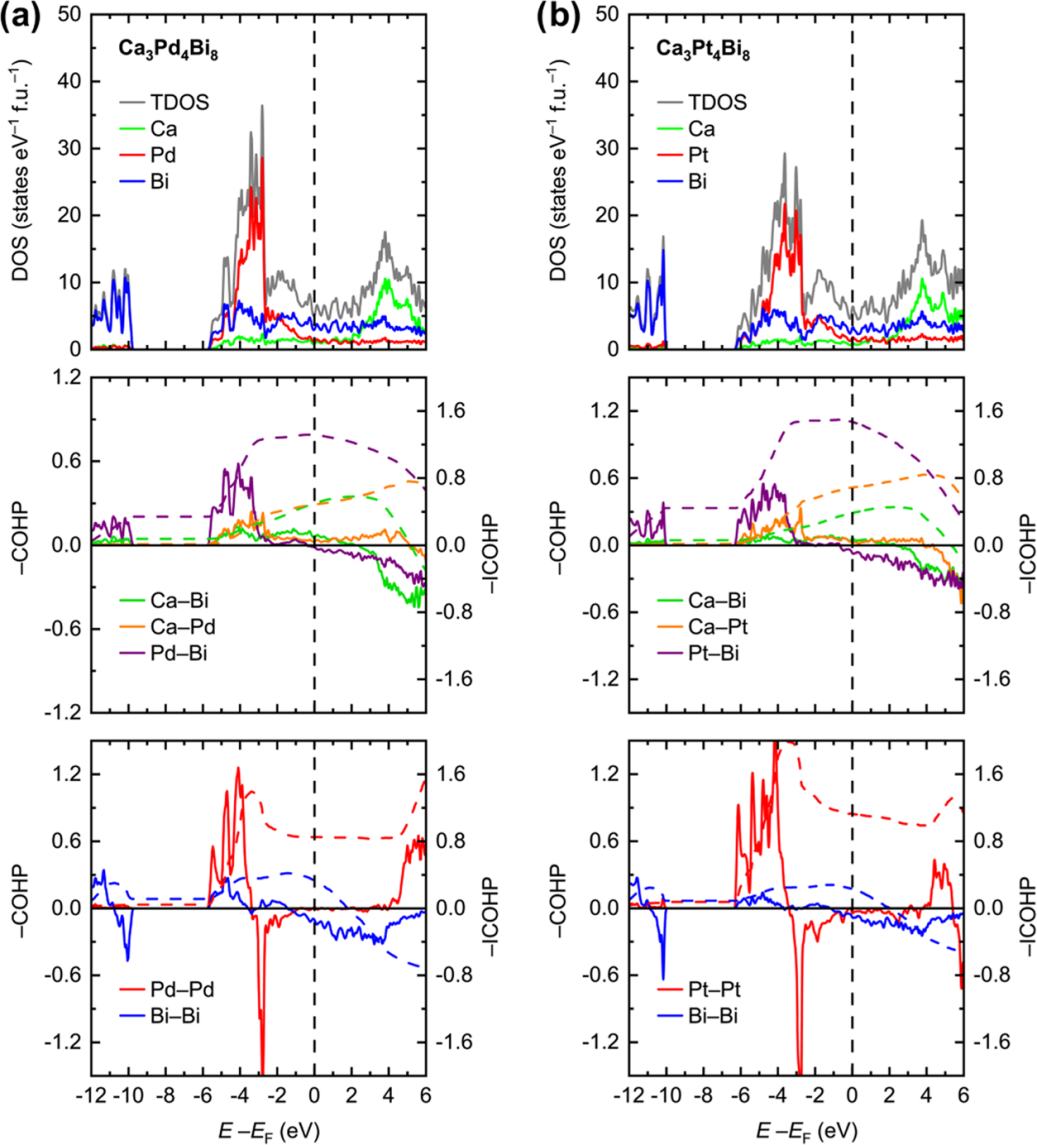
Total and projected electronic densities of states (DOS, top) and averaged crystal orbital Hamilton population curves (COHPs) for heteroatomic (middle) and homoatomic (bottom) interactions in Ca_3_Pd_4_Bi_8_ (a) and Ca_3_Pt_4_Bi_8_ (b).

Analysis of the electronic densities of states (DOS, Figure 7a,b top) for Ca_3_Pd_4_Bi_8_ and Ca_3_Pt_4_Bi_8_ suggests metallic behavior for both materials. Due to the electron delocalization and low local symmetry for most of the atoms (the majority of the atoms are located in the positions with the ..*m* site symmetry), considerable mixing of the electronic states is observed in a wide energy region around the Fermi level (*E*_F_). In the immediate vicinity of *E*_F_, the DOS is predominantly composed of the M(d) and Bi(p) states, where M is Pd or Pt, respectively. The M(d) states form a domain of localized character in the energy window −2 eV < *E* – *E*_F_ < −6 eV. The M(s) states are mainly located below the Fermi level and are rather delocalized. Well below the Fermi level, at *E* – *E*_F_ < −10 eV, the DOS is dominated by the Bi(s) states, mostly originating from the electron lone pairs on the Bi atoms. The presence of the lone pairs on the Bi atoms was previously noted for Ca_3_Pd_4_Bi_8_ based on the analysis of the electron localization function (ELF).^28^

The Ca electronic states are predominantly found above the Fermi level, indicating the cationic nature of the Ca species, although some states extend down to about *E* – *E*_F_ = −6 eV, which points toward incomplete charge transfer. Nevertheless, the general picture of electronic interactions in both Ca_3_Pd_4_Bi_8_ and Ca_3_Pt_4_Bi_8_ allows the description of their structures as consisting of the Ca^2+^ cations and [M_4_Bi_8_]^6–^ polyanions, in accordance with the notion proposed by Johrendt and Mewis for Ca_3_Pd_4_Bi_8_.^28^

The chemical bonding in Ca_3_Pd_4_Bi_8_ and Ca_3_Pt_4_Bi_8_ was examined with the aid of crystal orbital Hamilton population analysis (COHP, Figure 7a,b middle and bottom). The Ca–Bi, Ca–M, and M–Bi interactions (M = Pd or Pt) show exclusively bonding states below the Fermi level, while for the homoatomic M–M and Bi–Bi contacts, some occupation of antibonding states is also observed. The strongest interactions (per bond), as measured by the integrated COHP at *E*_F_, are the covalent M–Bi and the metal–metal M–M bonds. The M–Bi interactions are fully optimized as *E*_F_. Interestingly, the averaged Ca–M bond appears to be as strong as or even stronger than the Ca–Bi interaction. The latter is underoptimized at the Fermi level, which is anticipated owing to its partially ionic character. For the Ca–Bi interactions, as well as for the Ca–*M* contacts, the underoptimization is related to the availability of bonding states above the Fermi level. Due to the complex combination of bonding and antibonding states, not unusual for Bi-rich compounds,^39,44,51,52^ the averaged Bi–Bi interaction is comparably weak, although it is essentially optimized at *E*_F_.

**Figure 8:**
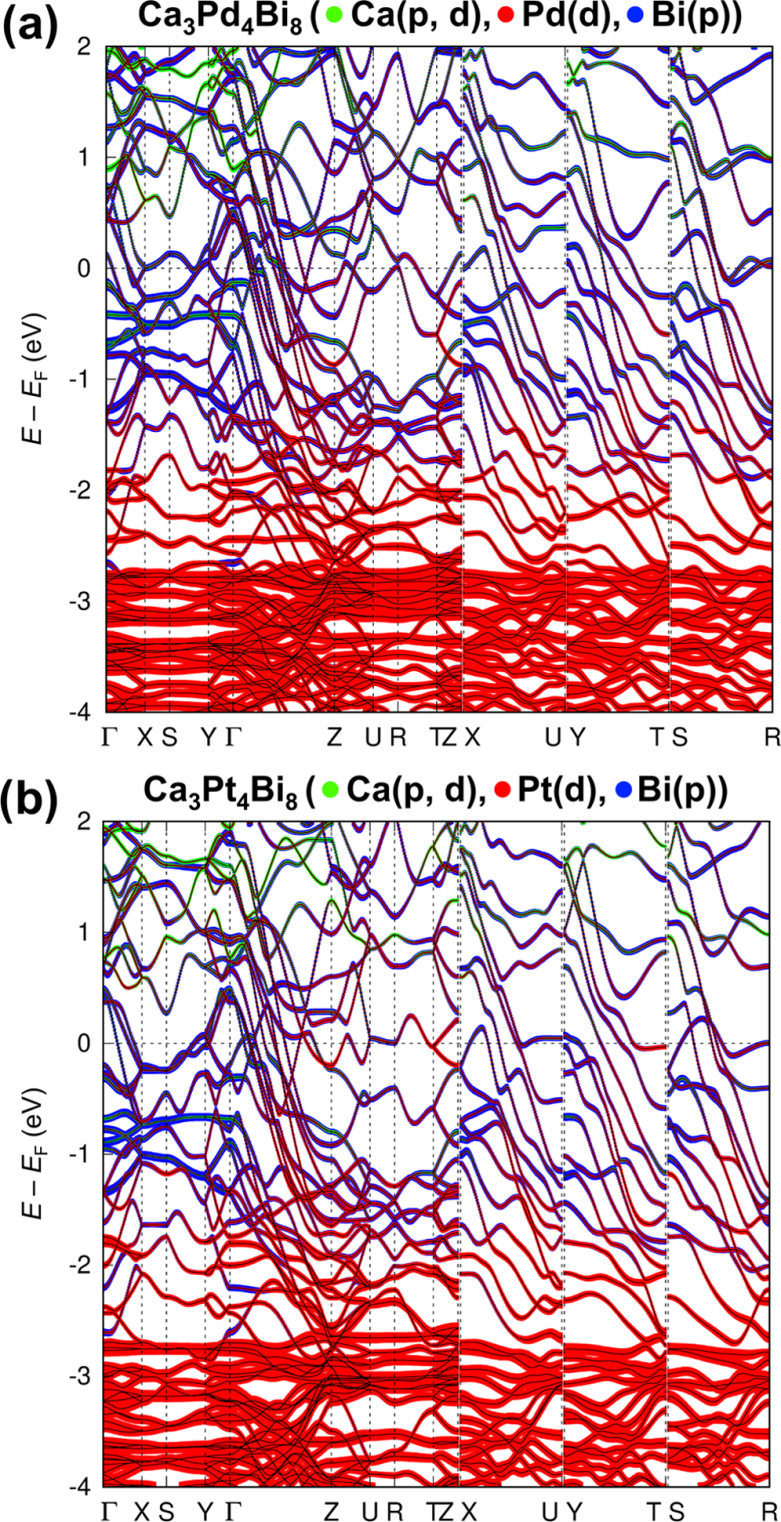
Electronic band structures and selected orbital projections (in “fat band” representation) for Ca_3_Pd_4_Bi_8_ (a) and Ca_3_Pt_4_Bi_8_ (b).

The band structures of Ca_3_Pd_4_Bi_8_ and Ca_3_Pt_4_Bi_8_ are given in Figure 8. From these images, it is evident that the metallic behavior of the two materials originates from their multiband character at the Fermi level. A noteworthy feature of both band structures is the high band dispersion along the paths Γ → Z, X → U, Y → T, and S → R in the momentum space, corresponding to the direction along [001] in the direct space, which suggests lower electron effective masses along the *c* axis. These steep bands exhibit predominantly the Bi(p) and M(d) character, with sizeable contributions from the Ca(p, d) states. Thus, the electronic structure reflects the quasi-one-dimensional nature of Ca_3_M_4_Bi_8_ with two kinds of interlinked polyhedral Pt−Bi chains running along the *c* direction (Figure 3, bottom).

Finally, we would like to address the question of charge distribution in Ca_3_M_4_Bi_8_. As was mentioned above, the ionic picture (Ca^2+^)_3_[M_4_Bi_8_]^6–^ appears to be a reasonable first approximation of the charge partitioning. However, the assignment of formal charges within the [M_4_Bi_8_]^6–^ polyanion is less straightforward. The high electronegativity of Pd and Pt calls into question the possible classification of Ca_3_M_4_Bi_8_ (M = Pd, Pt) as bismuthides, *i.e.*, compounds with anionic bismuth species. Indeed, the sizeable occupation of the M(d) states, observed in the projected DOS, points toward the anionic nature of the Pd and Pt species. A similar observation was previously made for Ca_3_Pd_4_Bi_8_, where analysis of the electronic state occupations pointed toward cationic and anionic nature for Ca and Pd, respectively, and essentially neutral character of the Bi atoms.^28^

The problem of charge assignment in compounds of electronegative noble metals with p-elements has been discussed before. For example, the ternary phases RE_3_Pt_4_Sn_6_ (RE = Pr–Dy) have been described as platinides rather than stannides, based on the Pt/Sn electronegativity differences.^53^ Similarly, the Al-rich compound Yb_2_Pt_6_Al_15_ has been identified as a platinide, rather than an aluminide.^54^

To evaluate the atomic charges in Ca_3_M_4_Bi_8_ (M = Pd, Pt), we used the approach of the quantum theory of atoms in molecules (QTAIM) and calculated the respective Bader charges,^55^ which depend only on the distribution of the total electron density, the latter being immediately available from our all-electron DFT calculations. The calculated Bader charges for Ca_3_Pd_4_Bi_8_ and Ca_3_Pt_4_Bi_8_ are presented in Table 4.

**Table 4:** Calculated Bader Charges for Ca_3_M_4_Bi_8_ (M = Pd, Pt)

atom	Ca_3_Pd_4_Bi_8_	Ca_3_Pt_4_Bi_8_
Ca1	+1.30	+1.22
Ca2	+1.27	+1.24
M1	–0.81	–1.16
M2	–0.74	–1.22
Bi1	+0.04	+0.21
Bi2	–0.28	–0.03
Bi3	–0.17	–0.09
Bi4	+0.02	+0.44

The Ca atoms in both compounds display a clear cationic character, in line with the charge partitioning discussed above, although the absolute values of the charges (around 1.2–1.3) are expectedly lower than the fully ionic limit of +2. Both symmetry-independent positions of the noble metal atoms are found to have negative Bader charges, which is not surprising taking into account their high electronegativity (2.20 and 2.28 for Pd and Pt, respectively, on the Pauling electronegativity scale^1^). The somewhat higher absolute values for the Pt Bader charges in comparison to those of Pd correlate well with the higher electronegativity of Pt, which in turn can be in part explained by the relativistic contraction of the Pt 6s state. This effect is responsible for stabilization of anionic Pt species in many compounds, including the ionic materials Cs_2_Pt and Cs_9_Pt_4_H.^56,57^ The charges on the Bi atoms, on the contrary, are rather small by absolute value: in both Ca_3_Pd_4_Bi_8_ and Ca_3_Pt_4_Bi_8_, the sites Bi1 and Bi4 exhibit weak cationic character, whereas the sites Bi2 and Bi3 possess low negative Bader charges. The magnitudes of the Bi charges change considerably upon going from M = Pd to Pt in Ca_3_M_4_Bi_8_, reflecting the interplay between the Pd/Pt electronegativity differences and subtle variations in the Bi–X (X = Ca, M, Bi) bonding interactions. For example, the longest Ca1–Bi1 bonding distance increases by about 0.022 Å upon going from Ca_3_Pd_4_Bi_8_ to Ca_3_Pt_4_Bi_8_, which probably reduces the electron density transfer onto the Bi1 atom, resulting in its higher positive charge in Ca_3_Pt_4_Bi_8_, and at the same time lowers the positive charge on the Ca1 atom, despite a shorter Ca1–M1 distance in the case of M = Pt. However, in general, identifying the origins of the Bi atomic charges is complicated due to the apparent presence of multicenter bonding and the possible effect of an extended coordination environment due to the metallicity of the studied compounds.

To sum up, the Bader charge distribution suggests that Ca_3_Pd_4_Bi_8_ and Ca_3_Pt_4_Bi_8_ cannot be classified as simple bismuthides. The highly negative charges on the Pd and Pt atoms call for their designation as palladide and platinide for Ca_3_Pd_4_Bi_8_ and Ca_3_Pt_4_Bi_8_, respectively. Following the convention of placing the anion at the end of the chemical formula, the compositions of the two compounds should be properly given as Ca_3_Bi_8_Pd_4_ and Ca_3_Bi_8_Pt_4_.

## Conclusions

The isostructural ternary compounds Ca_3_Pd_4_Bi_8_ and Ca_3_Pt_4_Bi_8_ have been successfully grown as mm-sized single crystals from a Bi flux using a sophisticated temperature program that allowed the growth of high-quality crystals in a process similar to Ostwald ripening.

Supported by electron structure calculations, Ca_3_Pd_4_Bi_8_ and Ca_3_Pt_4_Bi_8_ can be described as polar intermetallics featuring complex polyanions [M_4_Bi_8_]^6–^ (M = Pd, Pt). The polyanionic network comprises interlinked polyhedral M–Bi chains that propagate along the *c* direction in the orthorhombic crystal structure of Ca_3_M_4_Bi_8_ (M = Pd, Pt), while the Ca species occupy the channels formed in the polyanion between the M–Bi chains.

These crystal structural features result in quasi-one-dimensional electronic behavior, as suggested by our band-structure calculations. The structural stability is provided mainly by strong covalent M–Bi interactions in the polyanions, revealed in the analysis of the chemical bonding. Bader charges, calculated by integration of the total charge density in the atomic basins, point toward the presence of Ca cations with incomplete charge transfer and anionic character of the M sites in Ca_3_M_4_Bi_8_ (M = Pd, Pt), while the Bi atoms carry small charges with varying signs. For that reason, it is more appropriate to address the compounds as palladide and platinide, respectively, and the formulae should be rewritten as Ca_3_Bi_8_M_4_ (M = Pd, Pt).

Magnetization measurements indicate intrinsic diamagnetism of Ca_3_Pd_4_Bi_8_ and Ca_3_Pt_4_Bi_8_, with no signs of superconductivity down to 2 K. Electrical resistivity data suggest metallic behavior with dominating electron–phonon scattering and confirm the absence of superconductivity. Considering that many intermetallic compounds of Pd and Pt with Bi demonstrate superconductivity at even lower temperatures, further studies will be necessary to check if the ground state of Ca_3_Pd_4_Bi_8_ and Ca_3_Pt_4_Bi_8_ is superconducting.

## Supporting Information Available

Crystallographic data for CaPtBi, PXRD and magnetization data for Ca_3_Pt_4_Bi_8_ prepared in an Nb tube, visualization of Bi–Bi interatomic contacts in Ca_3_Pt_4_Bi_8_ (PDF)

## Accession Codes

CCDC 2165536–2165538 contain the supplementary crystallographic data for this paper. These data can be obtained free of charge via www.ccdc.cam.ac.uk/data_request/cif, or by emailing data_request@ccdc.cam.ac.uk, or by contacting The Cambridge Crystallographic Data Centre, 12 Union Road, Cambridge CB2 1EZ, UK; fax: +44 1223 336033.
