# Supplementary material for: Flux Growth, Crystal Structures, and Electronic Properties of the Ternary Intermetallic Compounds Ca3Pd4Bi8 and Ca3Pt4Bi8
Source: Inorg Chem. 2022 Jun 15;61(25):9756–66. doi: 10.1021/acs.inorgchem.2c01248 (PMC9490834; doi:10.1021/acs.inorgchem.2c01248)
Supplement: Supplementary file 1 — ic2c01248_si_001.pdf [file ic2c01248_si_001.pdf]

Supporting Information

Flux growth, crystal structures, and electronic properties of the ternary intermetallic compounds $Ca_3Pd_4Bi_8$ and $Ca_3Pt_4Bi_8$

Alexander Ovchinnikov[a] and Anja-Verena Mudring*[a,b]

[a] Department of Materials and Environmental Chemistry, Stockholm University, Svante Arrhenius väg 16 C, 10691 Stockholm, Sweden.

[b] Department of Chemistry, Aarhus University, Langelandsgade 140, 8000 Aarhus C, Denmark.

[*] Corresponding author: anja-verena.mudring@mmk.su.se

Table of Contents

Crystallographic data for CaPtBi……………………………………………………..………………..S2

PXRD and magnetization data for a $Ca_3Pt_4Bi_6$ sample prepared in Nb tube…...………………………S3

Bi–Bi interatomic contacts in $Ca_3Pt_4Bi_8$..…..…………………………………….……..………………..S4

Table S1. Refinement details and selected crystallographic data for CaPtBi (space group *Pnma*, room temperature, Mo Kα λ = 0.71073 Å, Z = 4)

Refined composition	CaPtBi
CCDC No.	2165538
fw/ g mol^{-1}	444.15
a/ Å	7.3722(15)
b/ Å	4.6998(9)
c/ Å	8.1167(16)
V/ Å^3	281.23
ρ_{calc}/ g cm^{-3}	10.49
$\mu_{MoK\alpha}$/ mm^{-1}	113.6
R_{int}	0.056
R_1 [$I > 2\sigma(I)$][a]	0.032
wR_2 [$I > 2\sigma(I)$][a]	0.071
R_1 [all data][a]	0.039
wR_2 [all data][a]	0.074
$\Delta\rho_{max,min}$/ e Å^{-3}	3.27, –2.66

[a]$R_1 = \sum||F_o| - |F_c||/\sum|F_o|$; $wR_2 = [\sum[w(F_o^2 - F_c^2)^2]/\sum[w(F_o^2)^2]]^{1/2}$, where $w = 1/[\sigma^2F_o^2 + (0.0369P)^2]$ and $P = (F_o^2 + 2F_c^2)/3$.

Table S2. Atomic coordinates and equivalent isotropic displacement parameters (Å^2) for CaPtBi

Atom	Site	x	y	z	U_{eq}[a]
Ca	4*c*	0.0077(3)	1/4	0.6989(3)	0.0174(6)
Pt	4*c*	0.29384(8)	1/4	0.41578(6)	0.0175(2)
Bi	4*c*	0.18702(7)	1/4	0.09004(5)	0.0150(2)

[a]U_{eq} is defined as one third of the trace of the orthogonalized U_{ij} tensor.

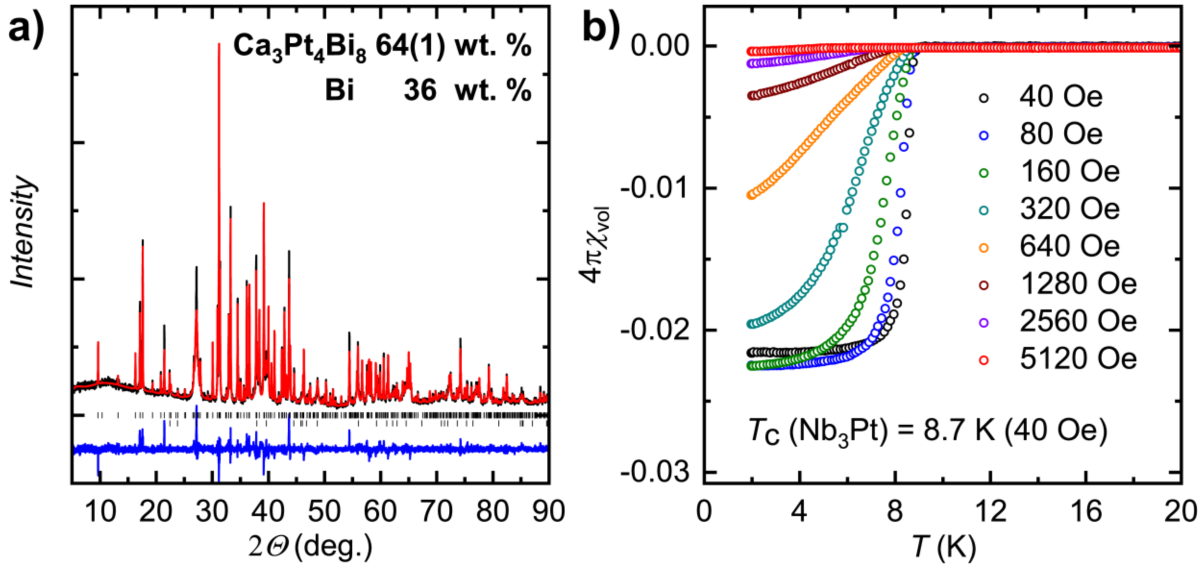

Figure S1. (a) Powder X-ray diffraction pattern (Cu Kα1) along with the corresponding Rietveld refinement for a $Ca_3Pt_4Bi_8$ sample prepared in a Nb tube. Experimental data, calculated profile, and difference curve are shown in black, red, and blue, respectively. Tick marks indicate the positions of the Bragg reflections for $Ca_3Pt_4Bi_8$ (upper row) and the major impurity, elemental Bi (bottom row). The phase fractions are indicated. (b) Low-temperature magnetic susceptibility for the same sample measured under different fields (zero-field-cooled regime). The apparent superconductivity below about 8.7 K is due to a Nb_3Pt impurity. The superconducting volume fraction estimated from the Meissner response is 2.2 %.

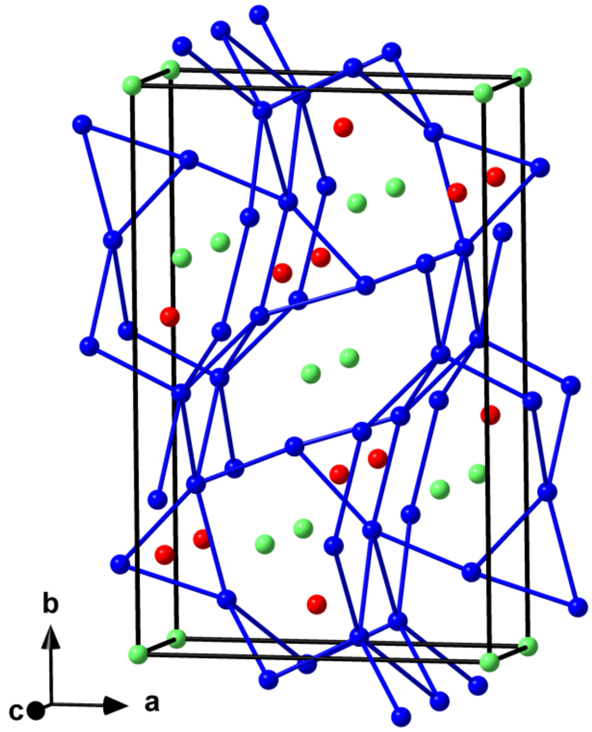

Figure S2. Network of Bi–Bi bonds ($d_{Bi–Bi} \leq 3.61$ Å) in $Ca_3Pt_4Bi_8$. Ca, Pt, and Bi atoms are shown in green, red, and blue, respectively. The unit cell is outlined in black.
